# Assessment of COVID-19 Anxiety Levels and Attitudes to COVID-19 Vaccine Among Pregnant Women in Poland

**DOI:** 10.1192/j.eurpsy.2023.1238

**Published:** 2023-07-19

**Authors:** K. Janik, K. Nietupska, G. Iwanowicz-Palus, M. Cybulski

**Affiliations:** 1 Department of Integrated Medical Care, Medical University of Bialystok, Bialystok; 2 Department of Obstetrics and Gynecology, Independent Public Healthcare Center in Sokolka, Sokolka; 3 Department of Development in Midwifery, Medical University of Lublin, Lublin, Poland

## Abstract

**Introduction:**

Approximately 15% of all pregnant women experience emotional changes that increase the risk of anxiety and depression, which can in turn adversely affect their health and their developing foetuses. There are literature reports indicating a significantly higher prevalence of anxiety and depressive symptoms in pregnant women during the COVID-19 pandemic than before the pandemic; however, their exact prevalence is currently unknown.

**Objectives:**

The aim of this study was to analyse and assess the prevalence of COVID-19 anxiety symptoms and to investigate the attitudes towards COVID-19 vaccination among pregnant women in Poland.

**Methods:**

The study included 288 women at different stages of pregnancy as the study group and 307 women of reproductive age (18-49 years) as the control group. A total of 595 women participated in the study. The study used a diagnostic survey method with a web-based questionnaire consisting of the author’s survey questionnaire and the following standardised tools: the Scale to Measure the Perception of SARS-CoV-2 Vaccines Acceptance (VAC-COVID-19 SCALE), the Fear of COVID-19 Scale (FCV-19S), the Drivers of COVID-19 Vaccination Acceptance Scale (DrVac-COVID19S) and the Coronavirus Anxiety Scale (CAS).

**Results:**

According to 25% of the study group and 42% of the control group, vaccination was safe and necessary, while 10% of pregnant women and 8% of women of reproductive age believed that the vaccine was dangerous. We found statistically significant differences between the groups for CAS (p = 0.025), DrVac-COVID19S (p = 0.00) or VAC-COVID-19 (p = 0.00). From the results, it can be seen that pregnant women scored significantly lower. Furthermore, both pregnant women and controls showed a high level of vaccine acceptance and positive attitudes towards it. The mean VAC-COVID-19 score was 44.26 in the control group and 41.44 in the study group, while the mean DrVac-COVID19S score was 51.25 for pregnant women and 55.85 for women of reproductive age. The mean CAS score was 0.61 in the pregnant group and 1.03 in the control group, respectively, suggesting a low level of anxiety associated with COVID-19. Other results on the severity of anxiety and fear of coronavirus were obtained using the FCV-19S. The mean score oscillated around 15 out of a possible 35, indicating moderate anxiety.

**Conclusions:**

Pregnant women generally showed moderate COVID-19 anxiety, but the results varied depending on the tool used. VAC-COVID-19 and DrVac-COVID19S scores confirmed the high level of vaccine acceptance among the women surveyed and positive attitudes towards it. There was a strong positive correlation between VAC-COVID-19 and DrVac-COVID19S. Insufficient knowledge of the effects or complications of the vaccine in the foetus were the most common reason for COVID-19 vaccine refusal among pregnant women.

**Disclosure of Interest:**

None Declared

